# E-cadherin Interacts With Posttranslationally-Modified AGO2 to Enhance miRISC Activity

**DOI:** 10.3389/fcell.2021.671244

**Published:** 2021-07-05

**Authors:** Jie-Ning Li, Hui-Lung Sun, Ming-Yang Wang, Pai-Sheng Chen

**Affiliations:** ^1^College of Medicine, Institute of Basic Medical Sciences, National Cheng Kung University, Tainan, Taiwan; ^2^Department of Medical Laboratory Science and Biotechnology, College of Medicine, National Cheng Kung University, Tainan, Taiwan; ^3^Department of Chemistry, Institute for Biophysical Dynamics, The University of Chicago, Chicago, IL, United States; ^4^Department of Surgery, National Taiwan University Hospital, Taipei, Taiwan; ^5^Department of Surgical Oncology, National Taiwan University Cancer Center, Taipei, Taiwan

**Keywords:** E-cadherin, AGO2, miRISC activity, protein stability, miRNA

## Abstract

MicroRNAs (miRNAs) are small non-coding RNAs which post-transcriptionally suppress target mRNAs expression and/or translation to modulate pathophyological processes. Expression and function of miRNAs are fine-tuned by a conserved biogenesis machinery involves two RNase-dependent processing steps of miRNA maturation and the final step of miRNA-induced silencing complex (miRISC)-mediated target silencing. A functional miRISC requires Argonaute 2 (AGO2) as an essential catalytic component which plays central roles in miRISC function. We uncovered a post-translational regulatory mechanism of AGO2 by E-cadherin. Mechanistically, E-cadherin activates ERK to phosphorylate AGO2, along with enhanced protein glycosylation. Consequently, the phosphorylated AGO2 was stabilized and ultimately resulted in induced miRISC activity on gene silencing. This study revealed a novel pathway for miRNA regulation through an E-cadherin-mediated miRISC activation.

## Graphical Abstract

Regulatory effect between E-cadherin and AGO2.

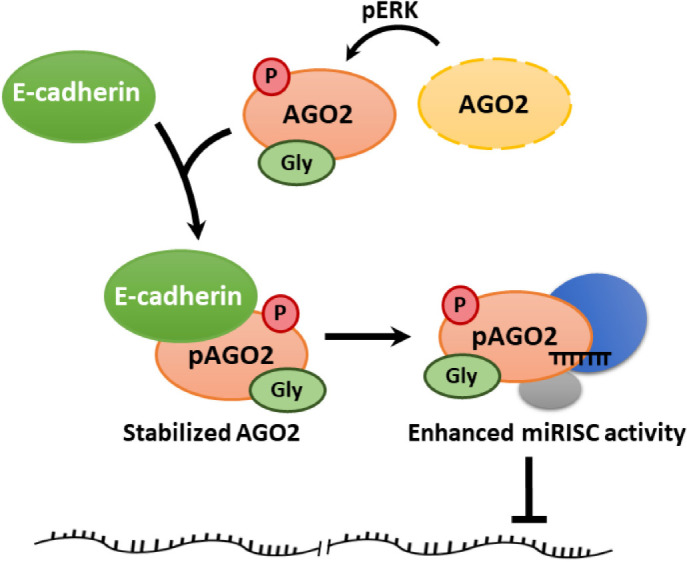

## Introduction

MicroRNAs (miRNAs) are small non-coding RNAs which suppress the expression of target genes. By base pairing to target mRNA 3′ UTR, miRNAs may induce degradation or translational inhibition of specific mRNAs and consequently results in the downregulation of target protein expression (Winter et al., 2009). MiRNA biogenesis is a two-step RNase-dependent cleavage process from nucleus to cytoplasm mediated by Drosha and Dicer, respectively (Winter et al., 2009). First, primary miRNAs (pri-miRNAs) are transcribed by RNA polymerase II and cleaved by Drosha/DGCR8 complex into precursor miRNAs (pre-miRNAs) in nucleus. Exportin-5 then transports pre-miRNAs to cytoplasm for Dicer-mediated cleavage into mature miRNA duplexes (Winter et al., 2009). Eventually, one strand of the resulting duplexes becomes guiding strand selectively loaded into miRNA-induced slicing complexes (miRISCs) for sequence-specific target mRNA recognition and suppression (Winter et al., 2009). MiRNAs are evolutionarily conserved mini-regulators expressed in animals and plants for maintaining proper biological functions. Thus, regulation/dysregulation of miRNA biosynthesis or modulation of miRISC activity eventually affects miRNA functions (Lin and Gregory, 2015; Gebert and MacRae, 2019). As a key component of miRISC, regulation of AGO2 results in altered miRISC activity (Treiber et al., 2019). Dephosphorylation of AGO2 on Tyr393 by protein tyrosine phosphatase 1B leads to reduced miRISC activity (Yang et al., 2014). Prolyl 4-hydroxylation of AGO2 on proline 700 (P700) is necessary for its stability and subsequently increases RNAi efficiency, that this phenomena is also observed in phosphorylation of AGO2 on serine 387 by p38 mitogen-activated protein kinase (Qi et al., 2008; Zeng et al., 2008; Johnston and Hutvagner, 2011). In addition to AGO2, numerous RNA-binding proteins are reported to regulate miRISC function (Yoo et al., 2011; Santhekadur and Kumar, 2020). For example, Tudor staphylococcal nuclease (TSN, also known as SND1) is an evolutionarily conserved protein with repeated nuclease domain, which has shown its function in post-transcriptional regulation. SND1 interacts with AGO2 in miRISC and facilitate mRNA degradation (Gutierrez-Beltran et al., 2016). PACT is an RNA-binding protein with dsRNA-binding domain, which is originally found as a protein activator of PKR (Redfern et al., 2013). PACT is not required for precursor-miRNAs processing but is essential for RNA-induced RNA interference. Interacting with both AGO2 and Dicer, PACT is a component of miRISC which facilitates its assembly (Lee et al., 2006). Another RNA-binding protein found to be involved in miRISC is fragile X mental retardation protein (FMRP). dFXR, Drosophila homolog of human FMRP, interacts with AGO2 and affects efficiency of miRISC in Drosophila S2 cells (Caudy et al., 2002; Didiot et al., 2009). In this study, we uncovered a novel mechanism of E-cadherin-regulated AGO2 protein phosphorylation and its impact on miRISC function.

## Results

### E-cadherin Upregulates and Interacts With AGO2 Protein

**FIGURE 1:**
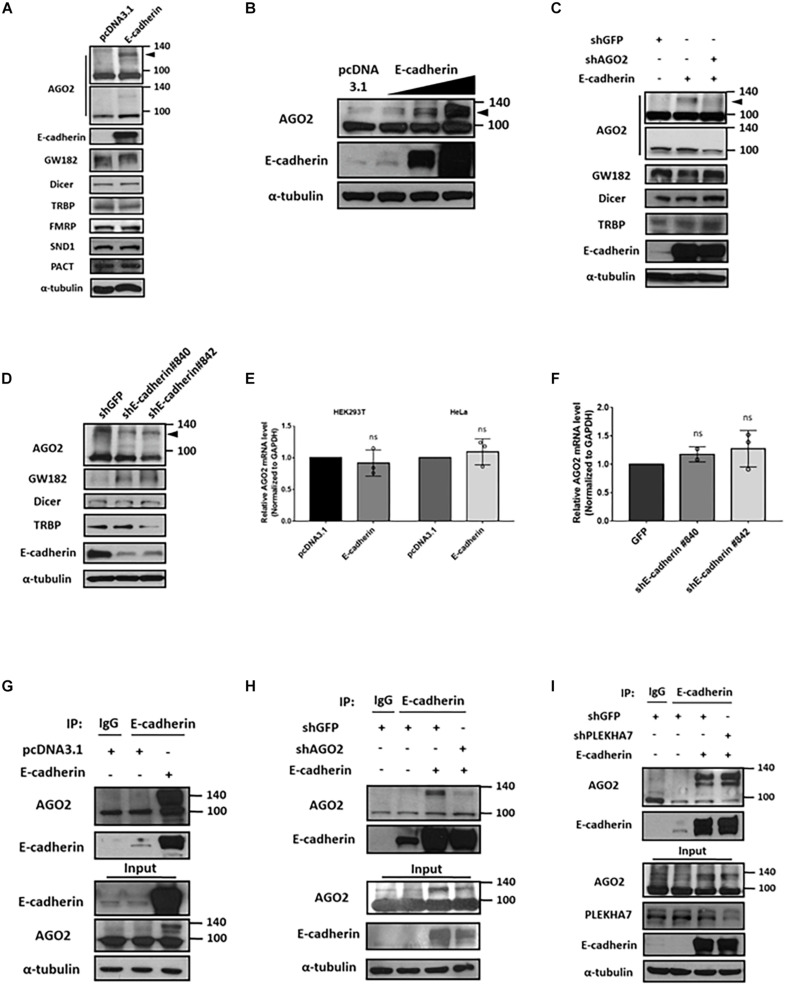
E-cadherin interacts with AGO2 and upregulates its protein expression. Effect of E-cadherin on protein expression of cytoplasmic miRNA biogenesis factors **(A-D)**. **(A)** AGO2, GW182, Dicer, TRBP, FMRP, SND1 and PACT protein expression were determined by western blot in E-cadherin-overexpressing HeLa cells. **(B)** AGO2 were determined by western blot in HeLa cells with sequential increase of E-cadherin overexpression. **(C)** Effects of AGO2 knockdown on protein expression of AGO2, GW182, Dicer and TRBP in E-cadherin-overexpressing HeLa cells. AGO2 was further knocked down by specific shRNA in E-cadherin-overexpressing HeLa cells. **(D)** E-cadherin was knocked down in MCF-7 cells using specific shRNAs. Protein expression of AGO2, GW182, Dicer and TRBP were determined by western blot. Effects of E-cadherin on mRNA expression of AGO2 **(E,F)**. **(E)** AGO2 mRNA expression was determined by real-time quantitative reverse-transcription PCR (qRT-PCR) in E-cadherin-overexpressing HeLa cells. **(F)** E-cadherin was knocked down in MCF-7 cells using specific shRNAs. mRNA expression of AGO2 was determined by qRT-PCR. Data were at least repeated in three independent experiments (mean ± SD) and statistically analyzed by two-tailed Student’s *t*-test **(E)** and one-way ANOVA **(F)**. **(G)** Interaction between E-cadherin and AGO2 in E-cadherin-overexpressing HeLa cells. Immunoprecipitation were performed using anti-E-cadherin antibody. **(H)** Effects of AGO2 knockdown on the interaction between E-cadherin and AGO2. AGO2 was knocked down by specific shRNA in E-cadherin-overexpressing HeLa cells. Immunoprecipitation was performed using anti-E-cadherin antibody. **(I)** Effects of PLEKHA7 knockdown on the interaction between E-cadherin and AGO2. PLEKHA7 was knocked down by specific shRNA in E-cadherin-overexpressing HeLa cells. Immunoprecipitation was performed using anti-E-cadherin antibody.

Since HeLa is reported as a E-cadherin-negative cell (Vessey et al., 1995), we verified the E-cadherin expression among HeLa and other cell lines including HEK293T and MCF-7 cells. The results showed that HeLa cells have undetectable E-cadherin expression level compared to MCF-7 cells and HEK293T (Supplementary Figure 1A). Therefore, we chose HeLa as our overexpression model for E-cadherin ectopically expressing experiments. In E-cadherin-overexpressing HeLa cells, the levels of cytoplasmic components including Dicer, TRBP, GW182, FMRP, SND1, and PACT were not affected (Figure 1A). However, we observed a slightly increased AGO2 expression in predicted molecular weight (∼100 kDa), and notably, an obvious induction of AGO2 at higher molecular weight (∼130 kDa) was detected (Figure 1A). Upon a genetic titration of E-cadherin overexpression, AGO2 at higher molecular weight was increased dose-dependently in HeLa cells (Figure 1B). Since E-cadherin expression of HEK293T cells are lower than MCF-7 cells, we also overexpressed E-cadherin in different titrated genetic levels. Again, the dose-dependently elevated AGO2 at higher molecular weight were also observed in HEK293T cells (Supplementary Figure 1B). The elevated AGO2 signal at higher molecular weight in E-cadherin-overexpressing HeLa cells was decreased after genetic knockdown by shRNA specifically targeting AGO2 (Figure 1C) or E-cadherin (Figure 1D), indicating that the high molecular weight AGO2 protein is upregulated by E-cadherin. Next, we wondered whether the RNA level of *AGO2* is altered under the genetic manipulations of E-cadherin expression. We determined the mRNA level of *AGO2* in E-cadherin overexpression (Figure 1E) or knockdown (Figure 1F) cells and found that *AGO2* mRNA level is not significantly changed (Figures 1E,F), suggesting that E-cadherin-mediated AGO2 regulation acts through a post-transcriptional manner, which led us to pursue the protein-protein interaction between E-cadherin and AGO2. Thus, we immunoprecipitated (IP) E-cadherin and observed the higher molecular weight AGO2 which was detected in the immunoprecipitation of E-cadherin IP (Figure 1G), the AGO2 signals were again further confirmed by shRNA specifically targeting AGO2 (Figure 1H). Since previous studies have reported that E-cadherin are both membranous and cytoplasmic protein (Bi et al., 2017; Bendardaf et al., 2019), we isolated cell membrane fraction with c-Met detection as a positive control for plasma membrane fractions to investigate the distribution of E-cadherin and AGO2. We found that both E-cadherin and AGO2 proteins are abundantly expressed in the plasma membrane fractions but also exist in cytoplasm fractions (Supplementary Figure 2A), which were consistent with our confocal images showing the colocalization of E-cadherin (green) and AGO2 (red) (Supplementary Figure 2B). Abovementioned results indicated that E-cadherin selectively upregulates and interacts with the high molecular weight AGO2. Since PLEKHA7 has been reported to be existed in cadherin complex and is associated with miRISC in polarized cells (Kourtidis et al., 2015), it is possible that the E-cadherin-AGO2 binding relies on PLEKHA7. However, the binding between E-cadherin and AGO2 remains unchanged in PLEKHA7-knockdown cells (Figure 1I), suggesting a PLEKHA7-independent E-cadherin protein interaction with AGO2.

### E-cadherin Enhances the ERK-Dependent Phosphorylation of AGO2

**FIGURE 2:**
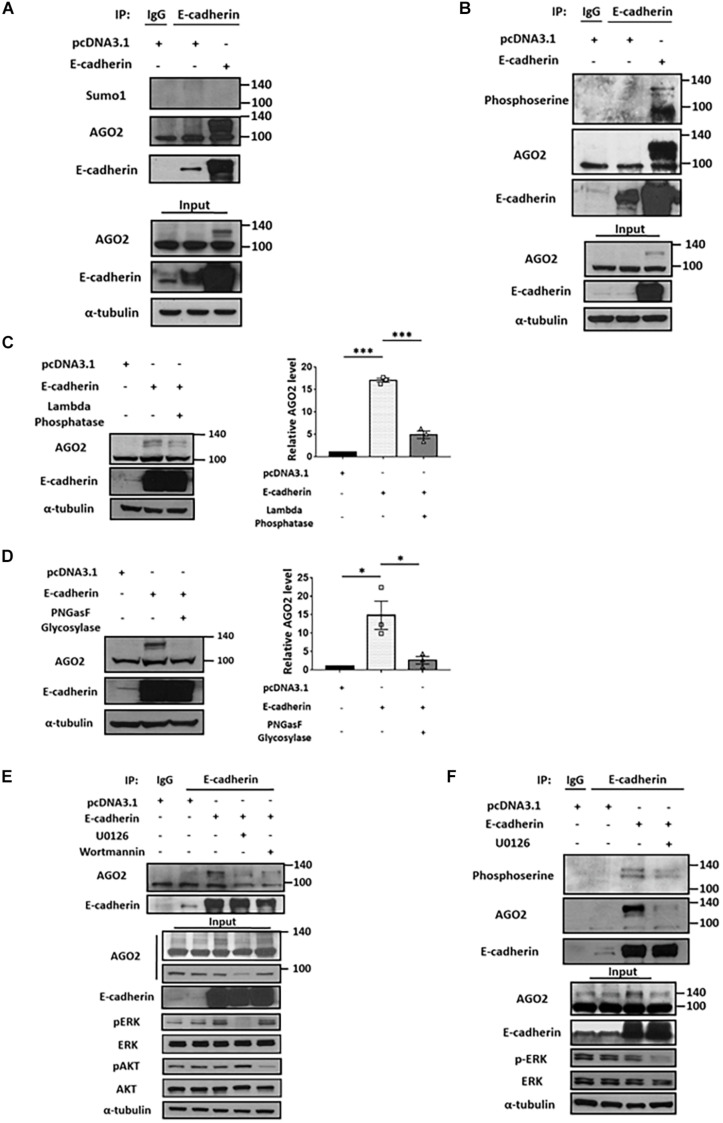
E-cadherin interacts with phosphorylated AGO2. Sumoylation and phosphorylation of AGO2 **(A,B)**. Detection of AGO2 sumoylation using anti-sumo1 antibody **(A)** or phosphorylation using anti-phosphoserine antibody **(B)** in E-cadherin-overexpressing HeLa cells were performed. Effect of lambda phosphatase **(C)** or PNGasF glycosylase **(D)** treatments on AGO2 expression in E-cadherin-overexpressing HeLa cells. Repeated data of panels **(C,D)** (upshifted band of AGO2) were quantitated using image J and repeated at least in three independent experiments (mean ± SEM) and statistically analyzed by one-way ANOVA. ^∗^*p* ≤ 0.05 and ^∗∗∗^*p* ≤ 0.001. **(E)** Effects of ERK or AKT inhibition on E-cadherin interaction with AGO2. 50 μM of U0126 and 300 nM of wortmannin were treated for 24 h in E-cadherin-overexpressing HeLa cells. **(F)** Effects of ERK inhibition on interaction between E-cadherin and phosphorylated AGO2. 50 μM of U0126 were treated for 24 h in E-cadherin-overexpressing HeLa cells. **(A,B,E,F)** E-cadherin was immunoprecipitated using anti-E-cadherin antibody and subjected for western blot analysis.

The molecular shift of protein mass is usually affected by posttranslational modifications (PTMs). Numerous PTMs may lead to increased molecular weight, such as phosphorylation, methylation, acetylation, glycosylation, ubiquitination and sumoylation (Jee and Lai, 2014). Currently, several types of AGO2 PTMs have been identified, including phosphorylation which enhances stability, modulates protein localization, and miRISC activity (Jee and Lai, 2014); ubiquitination (Bronevetsky et al., 2013; Smibert et al., 2013) or sumoylation which negatively regulates AGO2 stability (Sahin et al., 2014). Considering the level of observed molecular weight shift, we first determined whether the E-cadherin-interacting AGO2 is sumoylated. Immunoprecipitation of E-cadherin were performed and applied to western blot for the detection of AGO2 and sumo1 on the same gel. However, there is no detectable sumo1 signal for AGO2 protein, indicating that E-cadherin-interacting AGO2 is not modified by sumoylation (Figure 2A). In addition to sumoylation, phosphorylation of AGO2 have been discovered (Jee and Lai, 2014). Phosphorylation at S387 by AKT pathway alters AGO2 cellular localization and promotes miRISC activity (Bridge et al., 2017). The ERK-mediated S387 phosphorylation enhances AGO2 protein stability in neuron cells (Paradis-Isler and Boehm, 2018) and prevents AGO2 secretion into exosome (McKenzie et al., 2016). In addition, multi-site phosphorylation (S824-S834) by CSNK1A1 is necessary for efficient silencing of endogenous miRNA targets and fully efficient miRNA-mediated silencing (Golden et al., 2017). Y529 phosphorylation reduces AGO2 p-body localization (Mazumder et al., 2013). EGFR interacts with AGO2 under hypoxia leading to elevated Y393 phosphorylation and inhibit miRNA biogenesis (Shen et al., 2013). Having observed the accumulated AGO2 in E-cadherin-expressing cells, we proposed that E-cadherin interacts with phosphorylated AGO2, even though the shift caused by phosphorylation itself may not result in such an obvious molecular weight change, which was also observed by Nicolas et al. demonstrating a ∼30 kDa increase of AGO2 protein (Paradis-Isler and Boehm, 2018). Thus, we used anti-phosphoserine antibody and observed phosphoserine signal at the same molecular weight of E-cadherin-interacting AGO2 (Figure 2B). Supportively, the reduction of phosphorylated AGO2 at 130 kDa was further confirmed by lambda phosphatase treatment (Figure 2C). Usually, phosphorylation has been shown to induce a shift of only up to a few kDa. The unexpected shift of molecular weight (∼30 kDa) led us to study if there is any other modification exist. One of the possible PTMs, protein glycosylation, which may cause a more dramatic increase of molecular weight was then investigated. After using PNGasF glycosylase to remove the *N*-linked oligosaccharides, the signal of ∼130 kDa AGO2 was significantly decreased (Figure 2D). Together with these results suggest that both phosphorylation and glycosylation of AGO2 are enhanced by E-cadherin expression.

It is known that phosphorylation of AGO2 is induced by AKT and ERK pathways (Horman et al., 2013; McKenzie et al., 2016; Bridge et al., 2017). Therefore, we used U0126 and wortmannin to inhibit ERK and AKT pathway, respectively. In E-cadherin-overexpressing cells, the binding between E-cadherin and AGO2 was almost completely abolished by U0126 treatment (Figure 2E), suggesting that ERK dominantly contributes to AGO2 phosphorylation and E-cadherin interaction. The phosphoserine signal of AGO2 was decreased by U0126 treatment in E-cadherin-overexpressing cells, which confirmed the phosphorylation of AGO2 at higher molecular weight (Figure 2F). These results indicated a mechanism of ERK-dependent AGO2 phosphorylation for E-cadherin interaction.

### AGO2 Protein Is Stabilized by E-cadherin

**FIGURE 3:**
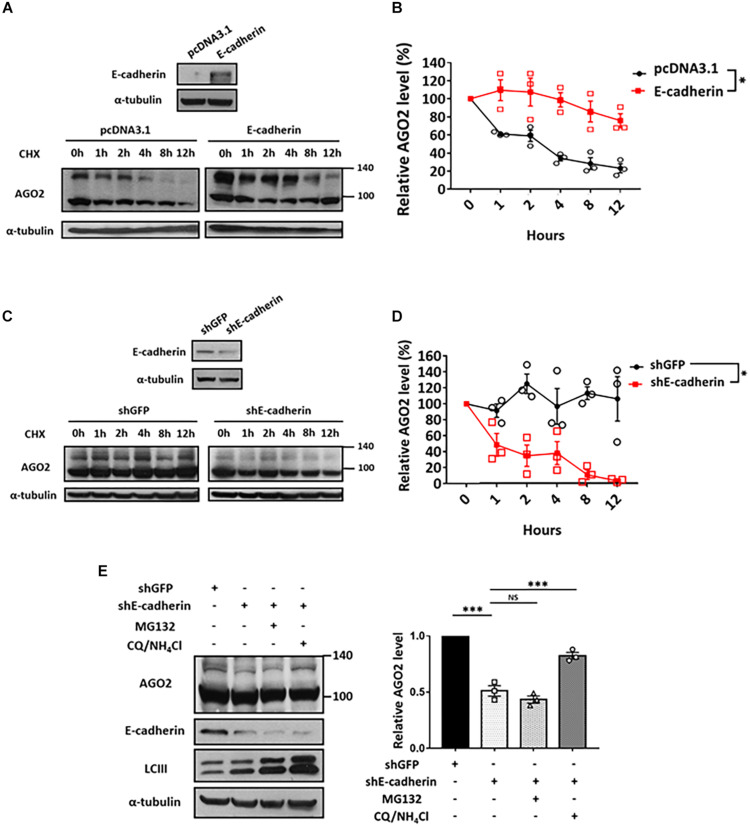
Lysosomal degradation of AGO2 protein is alleviated by E-cadherin. Determination of AGO2 protein stability in E-cadherin-overexpression and knockdown cells **(A-D)**. E-cadherin-overexpressing HeLa cells **(A,B)** or E-cadherin knockdown MCF-7 cells **(C,D)** were treated with 50 μg/mL of cycloheximide to block *de novo* protein synthesis and collected at indicated time points for determining AGO2 protein expression by western blot. **(B,D)** Degradation curves were plotted based on the quantification results of upshifted band of AGO2 from at least three independent experiments (mean ± SEM) and statistically analyzed by two-way ANOVA. ^∗^*p* ≤ 0.05. **(E)** Effect of MG132 and CQ/NH_4_Cl treatments on AGO2 expression in E-cadherin knockdown cells. 5 μM MG132 or 40 μM CQ/10 mM NH_4_Cl were treated for 24 h in E-cadherin knockdown cells. Repeated data of panel **(E)** (upshifted band of AGO2) were quantitated using image J and repeated at least in three independent experiments (mean ± SEM) and statistically analyzed by one-way ANOVA. ^∗∗∗^*p* ≤ 0.001. NS means no significance.

Since E-cadherin enhanced the expression of phosphorylated AGO2 without affecting its mRNA level (Figures 1A–D), we sought to determine the protein stability of AGO2. We performed cycloheximide treatment to block the *de novo* protein synthesis in either E-cadherin overexpression (Figures 3A,B) or knockdown (Figures 3C,D) cells and found that AGO2 protein exhibits enhanced stability in E-cadherin-overexpressing compared to control cells (Figures 3A,B). Similar results were also observed that protein degradation of AGO2 is significantly facilitated in E-cadherin knockdown cells (Figures 3C,D). These evidence indicated that E-cadherin upregulates AGO2 through prolonging its protein stability. In previews study, AGO2 has been found to be degraded by either selective autophagy or proteasome pathway (Qi et al., 2008; Gibbings et al., 2015; Paradis-Isler and Boehm, 2018). Therefore, to investigate the mechanism of E-cadherin-mediated AGO2 stabilization, we treated E-cadherin knockdown cells with MG132 or CQ/NH_4_Cl, the proteasome and lysosome inhibitors, respectively. Our results showed that the destabilized AGO2 protein in E-cadherin knockdown cells was restored by CQ/NH_4_Cl treatment, indicating that E-cadherin expression prevents AGO2 protein degradation through lysosome pathway (Figure 3E).

### E-cadherin Enhances miRISC-Mediated Gene Silencing

**FIGURE 4:**
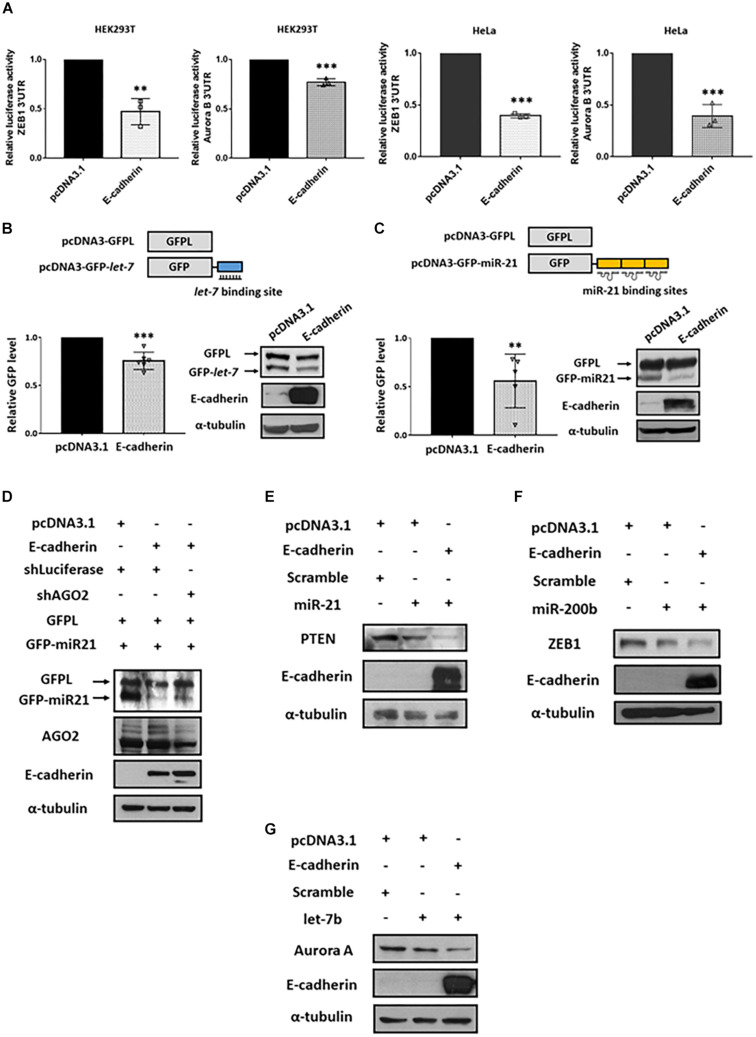
E-cadherin enhances miRISC-mediated gene silencing. Effect of E-cadherin on miRISC activity. **(A)** HEK293T or HeLa cells were transfected with ZEB1 or Aurora B 3′UTR reporter plasmids for 24 h. The luciferase activities were determined in E-cadherin-overexpressing HEK293T (purple) or HeLa (green) cells. E-cadherin-overexpressing HeLa cells were transfected with GFP-let-7 **(B)** or GFP-miR-21 **(C)** for 24 h to determine miRISC activity. **(D)** AGO2 was knocked down in E-cadherin-overexpressing HeLa cells. The cells were then transfected with GFP-miR-21 for determining miRISC activity. Cells were transfected with GFPL for transfection control. GFP and GFPL expression were determined by western blot using anti-GFP antibody. E-cadherin-overexpressing HEK293T or HeLa cells were transfected with miR-21 **(E)**, miR-200b **(F)**, or let-7b **(G)** for 24 h for analysis the expression of their target genes including PTEN, ZEB1, and Aurora A by western blot. Data are presented as mean ± SEM of at least three independent experiments and analyzed by two-tailed Student’s *t*-test. ^∗∗^*p* ≤ 0.01 and ^∗∗∗^*p* ≤ 0.001.

Argonaute 2 is the key factor essential for the activation and function of miRISC (Kobayashi and Tomari, 2016). Through miRNA-guided target mRNA recognition, miRISC suppresses target gene translation and/or mRNA degradation (Winter et al., 2009). Since E-cadherin stabilized AGO2 protein expression, we next investigated whether miRISC activity is consequently affected. First, we performed reporter activity assays using plasmids constructed with luciferase coding region and 3′ untranslated region (3′ UTR) of either ZEB1 (canonical target of miR-200b; Figure 4A, left) or Aurora B (canonical target of let-7b; Figure 4A, right) containing miRNA-binding sites (Gregory et al., 2008; Lal et al., 2009; Liu et al., 2013; Maki-Jouppila et al., 2015). Our results indicated that the 3′ UTR luciferase activities of both ZEB1 and Aurora B were reduced in E-cadherin overexpression cells (Figure 4A), suggesting that the enhanced miRISC activity induced by E-cadherin promotes the activities of endogenous miRNAs for target inhibition. To confirm whether these phenomenon act in miRNA-specific manner, we further transfected the plasmids harboring GFP coding sequence with either miR-21- (GFP-miR-21, Figure 4B) or let-7- (GFP-let-7, Figure 4C) binding region(s), while GFPL is a long form GFP used as transfection controls. In E-cadherin overexpression cells, either the expression of GFP-miR-21 (Figure 4B) or GFP-let-7 (Figure 4C) were inhibited, which confirms the induction of miR-21- and let-7-guided miRISC activities in gene suppression. Furthermore, in E-cadherin overexpression cells, the expression of GFP-miR-21 was restored after AGO2 was knocked down indicating the reduction of miRISC activity (Figure 4D). In support of these experiments using exogenous reporters as indicators for miRISC activities, we also determined the expression of endogenous miRNA-targeting genes including PTEN (canonical target of miR-21; Figure 4E; Meng et al., 2007; Zhang et al., 2010), ZEB1 (canonical target of miR-200b; Figure 4F; Tsai et al., 2017), and AuroraA (canonical target of let-7b; Figure 4G; Li et al., 2017). Our results showed that introduction of each miRNA mimics successfully inhibit the expression of corresponded target genes, and these miRISCs-mediated suppressive effects on endogenous gene expression were further enhanced by E-cadherin overexpression (Figures 4E–G). Together with these findings indicated that E-cadherin expression induces miRISC-mediated gene silencing.

## Discussion

E-cadherin has been studied for decades serving as a junctional protein which maintains cell-cell adhesion (Yu et al., 2019). In addition, its functions beyond structure protein have also been discovered. One of the studies related to our findings is that E-cadherin activates PI3K-AKT, MEK-ERK pathways and facilitates β-catenin/Wnt signaling to promote tumor progression (Yu et al., 2019). We demonstrated another molecular function of E-cadherin-mediated ERK activation in regulating AGO2 protein stability and the consequential enhanced miRISC activity (Figure 2E). On the other hand, PTMs of AGO2 including hydroxylation, sumoylation, ubiquitination, and phosphorylation were reported to regulate its protein stability (Smibert et al., 2013; Sahin et al., 2014; Paradis-Isler and Boehm, 2018). The hydroxylation of AGO2 at P700 and phosphorylation at S387 stabilizes its protein from proteasomal degradation, whereas sumoylation of AGO2 at K402 accelerates its degradation (Qi et al., 2008; Sahin et al., 2014). In addition, phosphorylation of AGO2 has been known to be induced by EGFR, ERK, AKT, and CSNK1A1 (Horman et al., 2013; Shen et al., 2013; McKenzie et al., 2016; Bridge et al., 2017; Golden et al., 2017), while our results showed that ERK plays a major role in E-cadherin-mediated AGO2 phosphorylation and the accompanied protein interaction. After properly processed by RNAse-dependent machinery, miRISC activity eventually controls the biological effects of mature miRNAs. The S387 and S824-S834 phosphorylation sites of AGO2 have been reported to regulate the landscape of protein interactome and miRISC formation (Horman et al., 2013; Bridge et al., 2017; Golden et al., 2017). Phosphorylation at S387 enhances GW182 and LIMD1 binding and miRISC activity (Horman et al., 2013; Bridge et al., 2017). Highly conserved S824-S834 residues were identified to contain phosphorylation sites for the induction of miRISC activity (Golden et al., 2017). There are also proteins identified for regulating miRISC activity (Liu et al., 2009; Yoo et al., 2011). Oncogene astrocyte elevated gene-1 (AEG-1) and staphylococcal nuclease domain containing 1 (SND1) have been reported to optimize miRISC gene silencing activity (Yoo et al., 2011). C3PO (component 3 promoter of RISC), a Mg^2+^-dependent endoribonuclease, is also reported to promote miRISC activation through removing siRNA passenger strand cleavage products (Liu et al., 2009). Here, we identified a novel function of E-cadherin in modulating miRISC activity through the interaction and stabilization of phosphorylated and glycosylated AGO2 and prevents its lysosomal degradation.

## Materials and Methods

### Western Blot

Cells were harvested and lysed by RIPA lysis buffer. Protein lysate were next applied to SDS-PAGE and transferred to PVDF membranes. After blocking using 5% non-fat milk in TBST for 60 min, membranes were washed by TBST and applied to primary antibodies including E-cadherin (BD Biosciences, 610181), AGO2 (GTX131422), Dicer (GTX130536), GW182 (Abclonal, A6115), Sumo1 (sc-9060), Phosphoserine (NB100-1953SS), GFP (GTX113617), ZEB1 (GTX105278), HMGA2 (GTX100519), and PTEN (GTX101025) in 4°C overnight. Membranes were washed 10 min for three times and applied for secondary antibodies for 60 min. Protein expression were visualized by ECL according to the manufacture’s protocols. ECL (Enhanced Chemiluminescent, PerkinElmer, Waltham, MA, United States). Image J was used for western blot quantification. The upshifted band of AGO2 was quantified. Image files were opened and the regions of upshifted band of AGO2 were selected and analyzed. Intensity of selected regions were showed in graph and the area of peaks were selected and calculated. The quantified results the upshifted band of AGO2 were normalized with α-tubulin. The collected data were subjected to statistical analysis.

### Immunoprecipitation

Cells were collected and lysed by NETN lysis buffer. Protein lysate was precleared using beads (Pierce Protein A Plus Agarose #22812) for 60 min in 4°C and supernatant was collected and incubated with antibodies including E-cadherin (BD Biosciences, 610181) in 4°C overnight. Beads were washed for two times with NETN lysis buffer and applied to western blot.

### RNA Extraction and Reverse Transcription Real-Time PCR

Total RNA was isolated using Trizol reagent according to manufacturer’s instructions. Trizol reagent was purchased from Invitrogen (Waltham, MA, United States). Reverse transcription was performed using 200 ng RNA. RNA was reverse transcribed complementary DNA (cDNA) using random primer (ReadyMade^TM^ Random Hexamers, IDT), reverse transcriptase, dNTPs and RNase inhibitors (Revert Aid First Strand cDNA Synthesis Kit, Thermo Fisher Scientific). Real-time PCR was performed by Applied Biosystem Step One Real-time PCR system using sybr green. Independent experiments at least three times were performed separately and GAPDH (Glyceraldehyde-3-Phosphate Dehydrogenase) was used as internal control.

### Transfection and Drug Treatment

Cells were seeded into dishes for attaching overnight. Plasmids were mix with transfection reagent (HyFectTM DNA Transfection Reagent, Leadgene) according to manufacturer’s instruction. Plasmids sources: pcDNA3-E-cadherin was obtained from Barry Gumbiner (Addgene plasmid # 45769) (Gottardi et al., 2001); GFP-L, GFP-let-7 and GFP-miR-21 were kindly provided by Dr. Hank Qi (Qi et al., 2008). pcDNA3-E-cadherin was transiently transfected for 24 h and applied to subsequent experiments. GFP-L and GFP-let-7 or GFP-miR-21 were transiently co-transfected for 24 h and cell lysates were subsequently harvested. miRNA mimics were transfected transiently for 48 h. 50 μM of U0126 (cat. 662005, Millipore), 300 nM of wortmannin (cat. 681675, Millipore), and 50 ug/ml of Cycloheximide (Cat.01810, Sigma) were used for treatment for indicated periods. Lambda phosphatase (sc-200312) was incubated with cell lysate at 30°C for overnight in final concentration 5 μM according to manufacturer’s instruction. PNGasF (P0704S) was incubated with cell lysate at 37°C for overnight according to manufacturer’s instruction.

### Lentiviral Knockdown

Knockdown experiments were performed using lentiviral shRNAs system from RNAi core (Academia Sinica, Taipei, Taiwan). HEK293T cells were transfected with three plasmids: packaging plasmid (pCMVΔR8.91), envelope plasmid (pMD.G), and shRNA plasmid (pLKO.1 shRNA) with proportion 10:10:1. Supernatant containing viral particles was collected and filtered with 0.22 μm filter after 24 h. Cells were infected with virus medium and polybrene for 24 h and selected with puromycin (1.5 μg/ml) for 48 h. shRNA sequence of AGO2: CCGGCCAGATTTCAAACTTGGATTTCTCGAGAAATCCAA GTTTGAAATCTGGTTTTT (RNAi core, Academia Sinica, Taipei, Taiwan).

### Luciferase Reporter Assay

Reporter plasmids were transfected into cells and cells were harvested after 24 h. Luciferase activity were assayed using Dual-Luciferase^®^ Reporter Assay System according to manufacturer’s instruction. Plasmids source: pCI-neo-RL-ZEB1 was a gift from Greg Goodall (Addgene plasmid # 35535) (Gregory et al., 2008); psiCHECK2-AURKB 3′ UTR was a gift from Judy Lieberman (Addgene plasmid #29475; Lal et al., 2009).

## Data Availability Statement

The raw data supporting the conclusions of this article will be made available by the authors, without undue reservation.

## Author Contributions

J-NL wrote the first draft of the manuscript. All authors contributed to conception and design of the study, and manuscript revision, read, and approved the submitted version.

## Conflict of Interest

The authors declare that the research was conducted in the absence of any commercial or financial relationships that could be construed as a potential conflict of interest.

## Supplementary Material

Supplementary Figure 1 Effect of E-cadherin on AGO2. **(A)** Expression of E-cadherin in HEK293T, HeLa and MCF-7 cells. **(B)** Effect of titrated overexpression of E-cadherin on AGO2 in HEK293T cells.

Supplementary Figure 2 Intracellular distribution of E-cadherin and AGO2. **(A)** Expression of E-cadherin and AGO2 in subcellular fractions. Plasma membrane and cytoplasmic fractions of MCF-7 cells were isolated and applied to western blotting analysis. C-Met expression was determined as a positive control for plasma membrane fractions. **(B)** E-cadherin colocalizes with AGO2. Confocal images of E-cadherin and AGO2. MCF-7 cells were co-stained with E-cadherin and AGO2 specific primary antibodies and examined by confocal microscopy.

## Abbreviations

miRNAs: MicroRNAs
pri-miRNAs: primary miRNAs
pre-miRNAs: precursor miRNAs
miRISC: miRNA-induced slicing complex
PTMs: posttranslational modifications
3′ UTR: 3′ untranslated region
