# Supplementary figures and images for: E-cadherin Interacts With Posttranslationally-Modified AGO2 to Enhance miRISC Activity
Source: Front Cell Dev Biol. 2021 Jul 5;9:671244. doi: 10.3389/fcell.2021.671244 (PMC8287304; doi:10.3389/fcell.2021.671244)

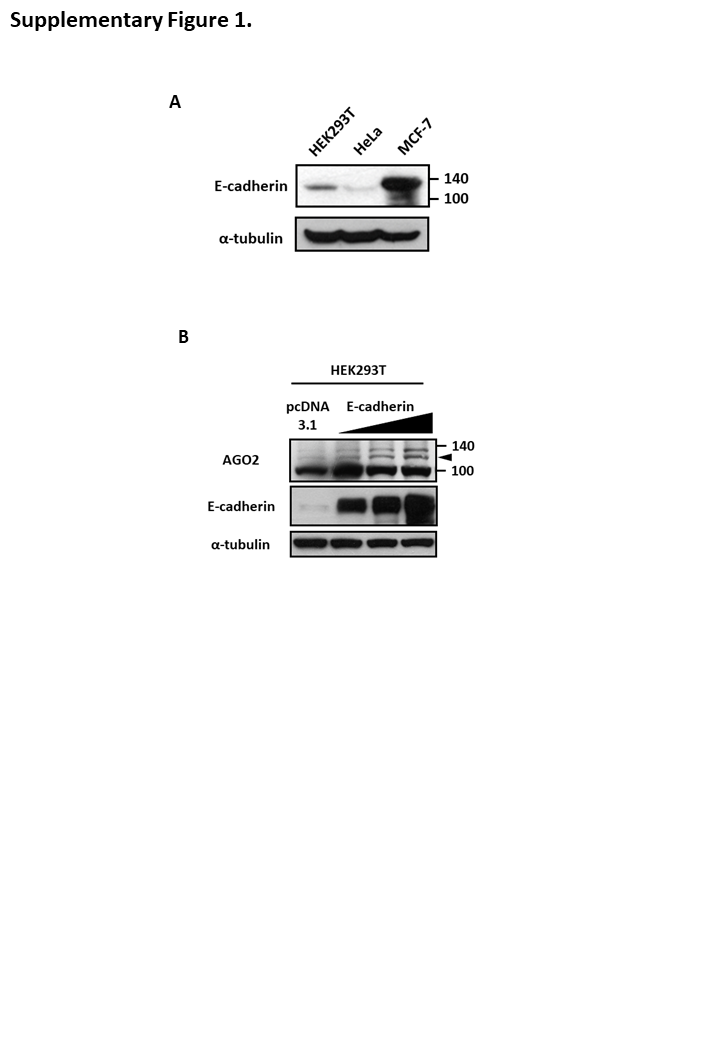

Supplement: Supplementary Figure 1 — Effect of E-cadherin on AGO2. (A) Expression of E-cadherin in HEK293T, HeLa and MCF-7 cells. (B) Effect of titrated overexpression of E-cadherin on AGO2 in HEK293T cells. [file Image_1.tif]

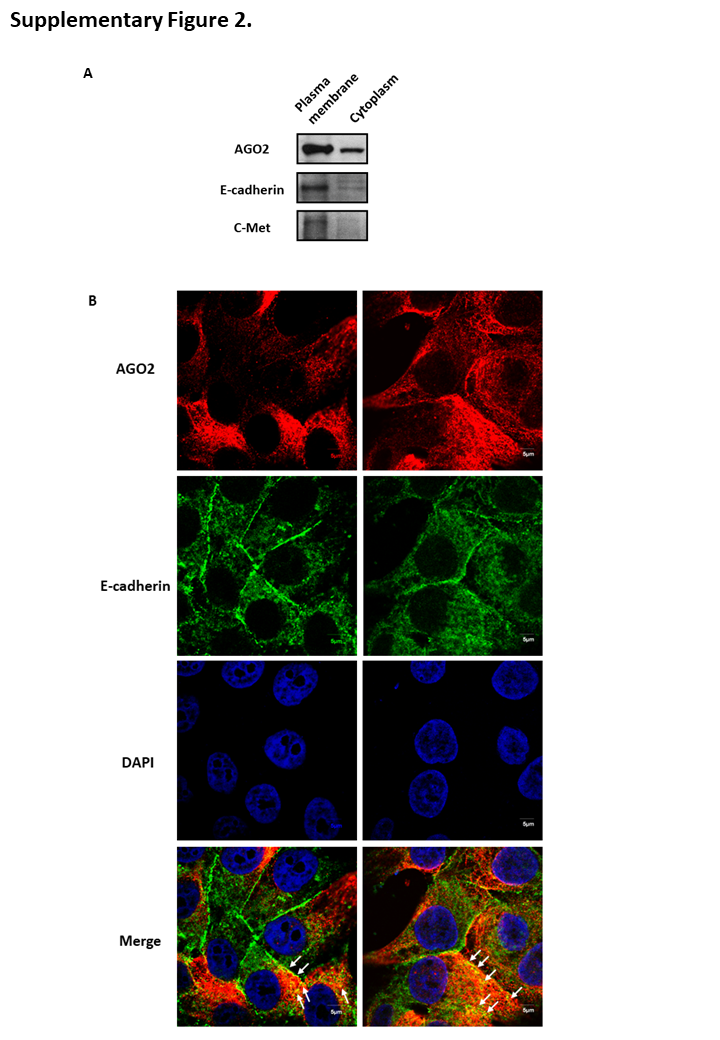

Supplement: Supplementary Figure 2 — Intracellular distribution of E-cadherin and AGO2. (A) Expression of E-cadherin and AGO2 in subcellular fractions. Plasma membrane and cytoplasmic fractions of MCF-7 cells were isolated and applied to western blotting analysis. C-Met expression was determined as a positive control for plasma membrane fractions. (B) E-cadherin colocalizes with AGO2. Confocal images of E-cadherin and AGO2. MCF-7 cells were co-stained with E-cadherin and AGO2 specific primary antibodies and examined by confocal microscopy. [file Image_2.tif]
